# Focus issue editorial: ecophysiology

**DOI:** 10.1093/plphys/kiag166

**Published:** 2026-03-30

**Authors:** Wagner L Araújo, Florian A Busch, Tracy Lawson, Christine Scoffoni, Xinguang Zhu, Agustin Zsögön

**Affiliations:** National Institute of Science and Technology on Plant Physiology Under Stress Conditions, Departamento de Biologia Vegetal, Universidade Federal de Viçosa, Viçosa, Minas Gerais 36570-900, Brazil; School of Biosciences and the Birmingham Institute of Forest Research, University of Birmingham, Birmingham, United Kingdom; School of Life Sciences, University of Essex, Colchester CO4 3SQ, United Kingdom; Department of Biological Sciences, California State University Los Angeles, 5151 State University Dr., Los Angeles, CA 90032, United States; Center of Excellence for Molecular Plant Sciences, Chinese Academy of Sciences, Shanghai 200032, China; National Institute of Science and Technology on Plant Physiology Under Stress Conditions, Departamento de Biologia Vegetal, Universidade Federal de Viçosa, Viçosa, Minas Gerais 36570-900, Brazil

Plant ecophysiology is an experimental discipline dedicated to understanding how plants respond to their physical, chemical, and biological environment. At its cores lies a fundamental question: how do plants function in real, dynamic, and often stressful environments? Addressing this question requires the integration of processes operating across multiple spatial and temporal scales, from molecules and cells to organs, whole plants, populations, and ecosystems, and from rapid responses occurring within seconds to long-term adjustments taking place over years or even decades. In the context of ongoing global changes, this integrative perspective has become more critical than ever. Climate uncertainty, increasing frequency of drought and heat waves, soil degradation, and intensifying biotic pressures are exposing plants to complex combinations of stresses that rarely occur in isolation. Under such conditions, understanding plant performance cannot rely solely on simplified, single-factor experiments conducted under controlled environments. Instead, ecophysiology provides the conceptual and methodological framework required to investigate how plants operate under realistic environmental scenarios.

This Focus Collection captures that integrative spirit very well. The papers gathered here span a broad conceptual and methodological range yet share a common goal: linking physiological mechanisms to ecological context. Across the collection, classical physiological processes, such as photosynthesis (Hammer et al. 2025; Li et al. 2025; Martin et al. 2025; Pan et al. 2025; Timm et al. 2025; Woodford et al. 2025), stomatal regulation (Daloso et al. 2026; Fan et al. 2025), carbon allocation (Chang et al. 2025; Vincent et al. 2025a; Li et al. 2026), water relation and hydraulics (Alongi et al. 2026; Bristow and Knipfer 2025; Cardoso et al. 2025; El Omari et al. 2026; He et al. 2025; Hirasawa et al. 2025; Pereira et al. 2025; Singh et al. 2026), nutrient use (Recalde et al. 2025), and stress (Deslauriers et al. 2025; Guardigli et al. 2026; Hammer et al. 2025; Hirasawa et al. 2025; Jené and Munné-Bosch 2025; Ma et al. 2025; Murphy et al. 2025; Pereira et al. 2025), are revisited through modern analytical approaches and emerging conceptual frameworks. By bridging mechanistic understanding with ecological complexity, these studies move beyond isolated mechanisms and instead illuminate how plants function under natural, complex, and rapidly changing environmental conditions.

A recurring theme across this Focus Collection is the reexamination of classical physiological processes through modern conceptual frameworks and advanced analytical tools. Several contributions revisit long-standing questions in plant metabolism, photosynthesis, and plant-pathogen interactions while placing them in the context of environmental acclimation and global change (eg Daloso et al. 2026; Thomazella et al. 2025; Vincent et al. 2025a). Another group of contributions focuses on central metabolism and its role in environmental acclimation. A particularly compelling synthesis by Timm et al. (2025) revisits photorespiration not as a wasteful process but as a central metabolic hub that supports plant acclimation to fluctuating environments. Their synthesis emphasizes the tight metabolic and redox integration of photorespiration with nitrogen assimilation, stress metabolism, and signaling pathways, reinforcing the view that processes once considered inefficient can in fact play essential roles in maintaining cellular homeostasis in fluctuating environments.

Several other studies in this collection further explore the coordination of photosynthesis, carbon allocation, and nutrient use efficiency (Hammer et al. 2025; Li et al. 2025; Martin et al. 2025; Murphy et al. 2025; Pan et al. 2025; Recalde et al. 2025; Woodford et al. 2025; Li et al. 2026). A research paper examining the declining CO_2_ fertilization effect in soybean reveals that changes in stomatal traits, photosynthetic efficiency, and transcriptional regulation under elevated CO_2_ constrain long-term productivity responses (Chang et al. 2025). Complementing this perspective, studies investigating nutrient limitation demonstrate that photosynthetic metabolism and nutrient acquisition are tightly integrated, with C_4_ plants responding differently to phosphate starvation than C_3_ plants (Krone et al. 2025). Together, these studies highlight the complex physiological tradeoffs underlying carbon gain and resource allocation under environmental change.

Water relations and plant hydraulics represent another central axis of plant ecophysiological function explored in this issue (Alongi et al. 2026; Bristow and Knipfer 2025; El Omari et al. 2026; He et al. 2025; Hirasawa et al. 2025; Martin et al. 2025; McAdam et al. 2025; Murphy et al. 2025). In a comparative analysis across plant lineages, Pereira and colleagues demonstrated that reductions in nighttime transpiration during drought arise through distinct regulatory mechanisms in ferns and seed plants, with hormonal control playing a prominent role in the latter (Pereira et al. 2025). Briefly, this work underscores the evolutionary diversity of plant water-use strategies and highlights how hydraulic regulation emerges from coordinated interactions between physiology, signaling, and environmental constraints. Another paper shows that under prolonged drought, the drought-resilient houseleek (*Sempervivum tectorum*) employs a quiescent-like survival strategy, maintaining low oxidative stress levels in leaves and using morphological adjustments to protect growing buds and preserve clonal offsets (Villadangos and Munné-Bosch 2025). This study reveals physiological and structural mechanisms that allow both parental plants and their vegetative offspring to survive extreme water deficit for many months without irreversible damage, shedding light on inherent drought resilience strategies in perennial species. Cardoso and colleagues authored a comprehensive update on plant hydraulic traits and crop performance under water limitation, where they synthesize current understanding of how hydraulic conductance, stomatal traits and related mechanisms influence crop yield during drought (Cardoso et al. 2025). This update also points out key knowledge gaps that future work should address to improve crop resilience under increasing soil dry-down and vapor pressure deficit conditions.

The collection also illustrates how ecophysiological insights increasingly rely on integrative and systems-level approaches (Mangaravite et al. 2025; Silvestre et al. 2026; Singh et al. 2026). For instance, by exploring how genome-scale metabolic models (GEMs)—which represent the full metabolic network of plants—can be constructed and used to predict metabolic responses to stressors like elevated CO_2_, drought, and high temperature, Mangaravite and colleagues discuss challenges in building accurate GEMs for plants, propose methodological solutions, and highlight how such models can generate hypotheses about metabolic adjustments under stress and guide breeding or engineering for climate resilience. Notably, multiomics analyses, gene regulatory network reconstruction, and computational modeling now allow researchers to bridge molecular processes with organismal performance and ecological outcomes. Such approaches are revealing emergent properties of plant physiological systems that cannot be understood through reductionist analyses alone.

Finally, several contributions emphasize the relevance of ecophysiology for agricultural sustainability and crop improvement (Pan et al. 2025; Siqueira et al. 2025; Vincent et al. 2025b). For instance, studies examining irrigation timing, metabolic responses to environmental cues and regulatory networks controlling growth and resource allocation illustrate how physiological knowledge can inform strategies to enhance crop resilience and productivity. In this context, watering time can significantly influence plant metabolism and biomass allocation in tomato, demonstrating how relatively simple management practices can exploit plant physiological plasticity to improve yield (Siqueira et al. 2025).

Taken together, the papers included in this Focus Collection Issue demonstrate that plant ecophysiology is evolving rapidly toward a multiscale and integrative science. By bridging molecular mechanisms with ecological context, these studies illuminate how plants function in complex environments and how physiological networks enable adaptation to environmental variability. As environmental change accelerates, such integrative perspectives will become increasingly essential not only for advancing fundamental plant biology but also for guiding strategies aimed at enhancing crop resilience, sustaining ecosystem function, and securing global food systems. Plant ecophysiology thus stands at a critical intersection of disciplines. By connecting physiology, ecology, molecular biology, and environmental science, it provides a powerful framework for understanding how plants will respond to the rapidly changing conditions that define the Anthropocene.

## Data Availability

No data was generated in the creation of this editorial.
